# Supplementary figures and images for: Thinking small: Next-generation sensor networks close the size gap in vertebrate biologging
Source: PLoS Biol. 2020 Apr 2;18(4):e3000655. doi: 10.1371/journal.pbio.3000655 (PMC7117662; doi:10.1371/journal.pbio.3000655)

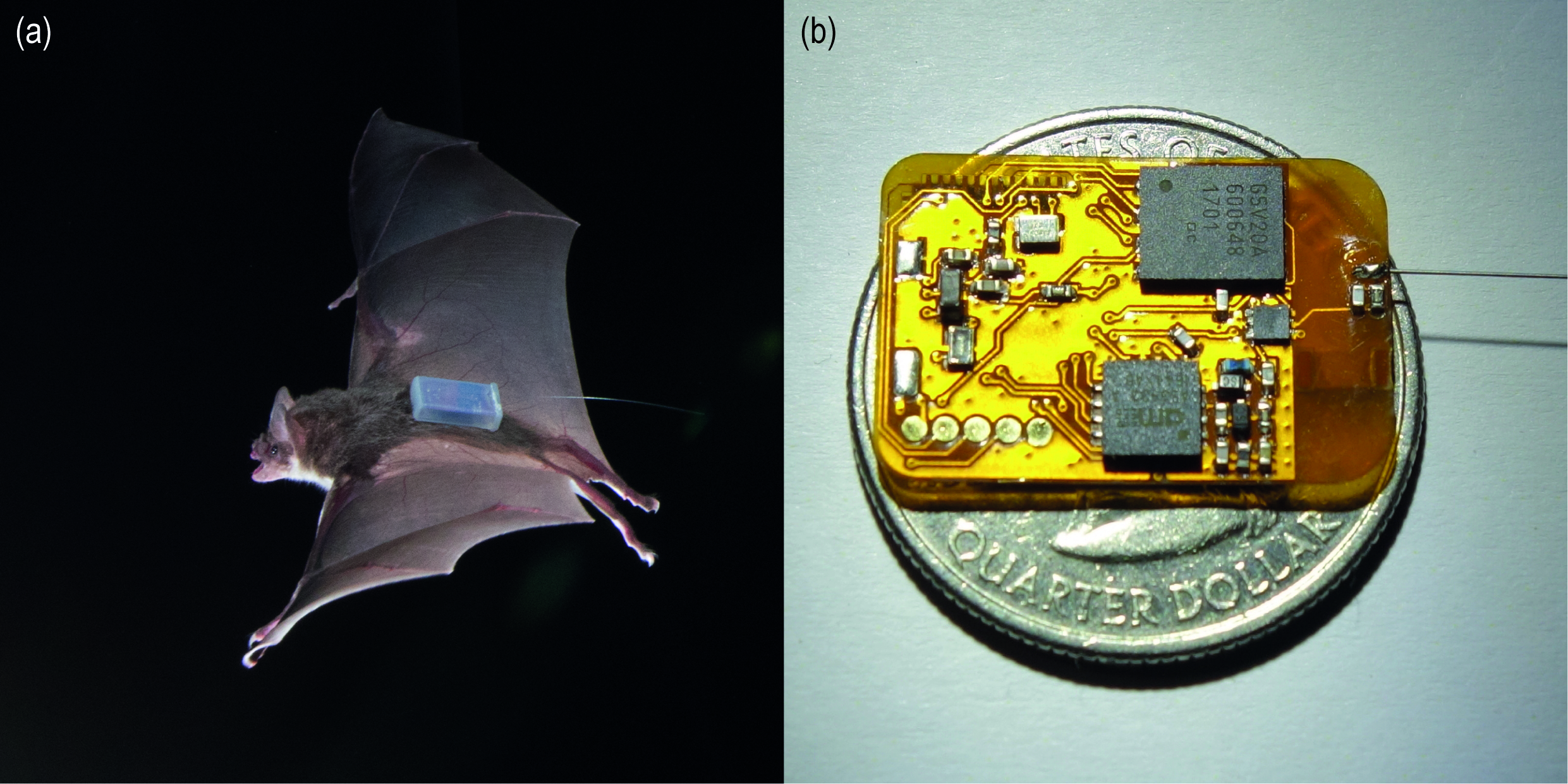

Supplement: S1 Fig — (A) Common vampire bat (Desmodus rotundus) carrying a mobile node housed in a plastic case; (B) bare mobile node on a quarter US dollar coin for comparison of size. Credits: Sherri and Brock Fenton (A), Peter Wägemann (B). (TIF) [file pbio.3000655.s001.tif]

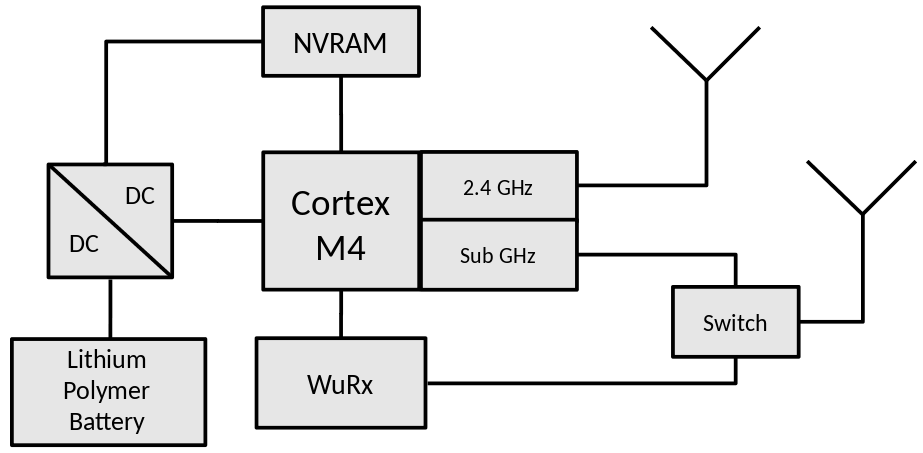

Supplement: S2 Fig — The assembled node is connected to a lithium polymer battery, which is folded to lie in parallel to the mobile node. A 3D printed plastic case including lid can be used as housing (see S1 Data; a hole for the whip antenna must be added manually). Specific components: DC/DC (DC-to-DC converter) = TPS82740 (by Texas Instruments); NVRAM (nonvolatile random-access memory) = FM25V20A (by Cypress Semiconductor Corp.); Cortex M4 / sub-GHz / 2.4 GHz (microcontroller) = EFR32FG1P133F256GM48 (by Silicon Labs); WuRx (wake-up receiver) = AS3933 (by ams), Switch (single-pole double-throw switch) = SKY13350 (by Skywork Solutions Inc.); antenna 2.4 GHz = AMCA31-2R450G-S1F-T (by Abracon); antenna sub-GHz = whip antenna. (TIF) [file pbio.3000655.s002.tif]
