# Supplementary material for: Thinking small: Next-generation sensor networks close the size gap in vertebrate biologging
Source: PLoS Biol. 2020 Apr 2;18(4):e3000655. doi: 10.1371/journal.pbio.3000655 (PMC7117662; doi:10.1371/journal.pbio.3000655)
Supplement: S1 Table — Description of hardware components, communication, data types, and software tasks considered for the energy model. (DOCX) [file pbio.3000655.s003.docx]

| **Keyword** | **Description** |
| --- | --- |
| **Hardware** | |
| Mobile node | Animal-borne sensor node. |
| Ground node | Multifunctional ground-based sensor nodes. Depending on the ground node configuration, it receives data from mobile nodes, triggers transmission of localization packets at the mobile node, or triggers the transmission of presence signals at the mobile node. |
| Localization node | Sensor node containing a dual-band antenna for high-resolution positioning of mobile nodes. Arranged in a tracking grid. |
| Long-range receiver | Long-range telemetry-dedicated sensor node for receiving long-range bursts. |
| **Communication and data types** | |
| Ground-node beacon | A packet sent by a ground node that triggers action on the mobile node (e.g., data download). |
| Mobile-node beacon | A packet sent by a mobile node. It contains a wakeup sequence which wakes other mobile nodes from standby. |
| Long-range burst | A packet embedded in the wakeup sequence of the mobile-node beacon for data transmission to a long-range receiver. |
| Localization packet | A packet sent by a mobile node to localization nodes for high-resolution tracking. Transmission is initiated by a ground-node beacon. |
| Presence signal | A packet sent by a mobile node to indicate the presence of a bat in range of a ground node. Transmission is initiated by a ground-node beacon. |
| **Software tasks on the mobile node** | |
| (i) standby | An idle state where only mandatory devices are active (e.g., wakeup receiver). The idle state is terminated by the reception of a wakeup. |
| (ii) sending beacons | Sending a mobile-node beacon to wake nearby mobile nodes from standby. Nearby mobile nodes immediately send their IDs in return. |
| (iii) receiving beacons | If the wakeup receiver receives a wakeup sequence, the conventional receiver is activated to receive the IDs of nearby mobile nodes (encoded in the mobile-node beacons). |
| (iv) observing ground-node availability | Every 2 s the receiver is activated to check whether ground-node beacons are received. |
| (v) transmitting data to a ground node | If beacons of a download-dedicated ground node are received, the transmitter is activated and data download is initiated. |
| (vi) sending localization packets | If beacons of a tracking-dedicated ground node are received, localization packets are sent (duty cycle 8/s; 868MHz & 2.4GHz) |
| (vii) sending presence signals | If beacons of a ground node are received, a presence signals is send |
