# Supplementary material for: Thinking small: Next-generation sensor networks close the size gap in vertebrate biologging
Source: PLoS Biol. 2020 Apr 2;18(4):e3000655. doi: 10.1371/journal.pbio.3000655 (PMC7117662; doi:10.1371/journal.pbio.3000655)
Supplement: Attachment — Submitted filename: reviewers comments_v2.docx [file pbio.3000655.s005.docx]

Dear Dr Ripperger,

Thank you very much for submitting your manuscript "Thinking small: next-generation sensor networks close the size gap in vertebrate biologging" for consideration as a Methods and Resources at PLOS Biology. Your manuscript has been evaluated by the PLOS Biology editors, an Academic Editor with relevant expertise, and by three several independent reviewers.

You'll see that all of the reviewers are broadly positive, and in light of the reviews, we're pleased to offer you the opportunity to address the comments from the reviewers in a revised version that we anticipate should not take you very long. We will then assess your revised manuscript and your response to the reviewers' comments and we may consult the reviewers again.

IMPORTANT: The Academic Editor notes that the reviewers request explicit qualitative (revs #1 and #2) and quantitative (rev #3) comparisons with other existing technologies (e.g. zebraNet) to better show the novelty, advantages, and disadvantages of your described methodology (Rev #1's suggestion to tabulate the comparison seems helpful). The Academic Editor also agrees with Rev#3's arguments that the BATS acronym is unhelpful in summarising either the content or applicability of the method, and recommends that you avoid it.

Dear editors,

We thank the editor and the three reviewers for their very helpful suggestions. We have responded to each comment in blue below. We have marked quoted text from the manuscript in *italics* below. Note that the line numbers refer to the tracked-changes version of the manuscript, not to the clean manuscript file. We are grateful for this careful consideration of our manuscript. Thanks to these points, we feel that the revised version reads much stronger, with increased clarity and impact.

On behalf of all co-authors,

Simon Ripperger

Our response to the short summary provided by the Academic Editor:

Qualitative comparisons to other systems:
We agree with the editor and the reviewers that a comparison across systems in form of a table will be very helpful to the reader to get a first impression of the capability of the presented system and the differences to other systems. We now present a comparison among systems suited to monitor small animals (the animal-borne tag should be 2.5 g or lighter) and which can track locations and/or encounters. We limited the comparison to lightweight animal-borne loggers because this paper focusses on tracking small animals and we wanted to provide a comprehensive overview. We contacted users or developers of all systems by phone or email (these individuals are identified in the table) to provide first-hand experiences, which go beyond what is found in the published papers. Several systems have only been published during the past 2 years, making this overview particularly timely. We did not include ZebraNet (as suggested by one of the reviewers), since this system (published first in 2004) can only be used for large animals such as ungulates (500g tag weight) and therefore does not fall within the scope of this manuscript. The new table now addresses many of the points raised below by the reviewers.

Quantitative comparisons to EncounterNet:

EncounterNet is in its proximity-sensing functionality similar to the system described in our paper. The reviewer suggested comparing the data quality provided by both by resampling our dataset based on sampling intervals used by EncounterNet (20s). While this approach seems informative, it does not really compare the systems themselves but rather 2 settings of the systems. I contacted Iris Levin who studied swallows with EncounterNet. She chose an inter-pulse interval of 20 s as a trade-off between temporal data resolution and runtime of the tags which got her 15 h of active logging. The inter-pulse intervals are configurable in both EncounterNet and the presented system. This information is not easily obtained from the published papers, I obtained this information from Iris Levin directly. In both cases, we tailor the sampling rate to our specific research question. The main difference is that our presented system uses an energy-saving wake-up receiver and ultra-low-power communication protocols so it achieves about 20 times the runtimes at the same sampling rates.

We therefore chose a slightly different approach to make an intuitive qualitative comparison among the systems based on simulated data from our energy model. We compared the runtimes of both systems with the same sampling interval. At a 20 s inter-pulse interval, we get 15 h in EncounterNet vs 321 h in our system. We added this information to the discussion (see below)).

This runtime limitation forces EncounterNet users to choose lower sampling rates, which means that potentially valuable information gets lost (as suspected by reviewer 3). We now also highlight this qualitative difference by referring to a paper of ours where we revealed maternal guidance in bats, based on short meetings of only few seconds, which might easily go unseen when using EncounterNet (see details below).

Regarding the acronym ‘BATS’: We agree that ‘Broadly Applicable Tracking System’ is pretty generic. In German, BATS stands for Betriebsadaptive Tracking-Sensorsysteme, which is an accurate description of the system but does not translate well to English. As suggested, we do not use ‘broadly applicable tracking systems’. Only in the methods section we make clear that these developments were enabled by the BATS-initiative so that people can link this manuscript to related work (we mention the German title and an English translation here). In the remaining main text we now use ‘the presented wireless biologging system’ or ‘the presented system’ where we used ‘BATS’ in the first version of the manuscript.

Your revisions should address the specific points made by each reviewer. Please submit a file detailing your responses to the editorial requests and a point-by-point response to all of the reviewers' comments that indicates the changes you have made to the manuscript. In addition to a clean copy of the manuscript, please upload a 'track-changes' version of your manuscript that specifies the edits made. This should be uploaded as a "Related" file type. You should also cite any additional relevant literature that has been published since the original submission and mention any additional citations in your response.

Please note while forming your response, if your article is accepted, you may have the opportunity to make the peer review history publicly available. The record will include editor decision letters (with reviews) and your responses to reviewer comments. If eligible, we will contact you to opt in or out.

Before you revise your manuscript, please review the following PLOS policy and formatting requirements checklist PDF: <http://journals.plos.org/plosbiology/s/file?id=9411/plos-biology-formatting-checklist.pdf>. It is helpful if you format your revision according to our requirements - should your paper subsequently be accepted, this will save time at the acceptance stage.

Please note that as a condition of publication PLOS' data policy (<http://journals.plos.org/plosbiology/s/data-availability>) requires that you make available all data used to draw the conclusions arrived at in your manuscript. If you have not already done so, you must include any data used in your manuscript either in appropriate repositories, within the body of the manuscript, or as supporting information (N.B. this includes any numerical values that were used to generate graphs, histograms etc.). For an example see here: <http://www.plosbiology.org/article/info%3Adoi%2F10.1371%2Fjournal.pbio.1001908#s5>.

For manuscripts submitted on or after 1st July 2019, we require the original, uncropped and minimally adjusted images supporting all blot and gel results reported in an article's figures or Supporting Information files. We will require these files before a manuscript can be accepted so please prepare them now, if you have not already uploaded them. Please carefully read our guidelines for how to prepare and upload this data: <https://journals.plos.org/plosbiology/s/figures#loc-blot-and-gel-reporting-requirements>.

Upon resubmission, the editors assess your revision and assuming the editors and Academic Editor feel that the revised manuscript remains appropriate for the journal, we may send the manuscript for re-review. We aim to consult the same Academic Editor and reviewers for revised manuscripts but may consult others if needed.

We expect to receive your revised manuscript within one month. Please email us ([plosbiology@plos.org](mailto:plosbiology@plos.org)) to discuss this if you have any questions or concerns, or would like to request an extension. At this stage, your manuscript remains formally under active consideration at our journal; please notify us by email if you do not wish to submit a revision and instead wish to pursue publication elsewhere, so that we may end consideration of the manuscript at PLOS Biology.

When you are ready to submit a revised version of your manuscript, please go to <https://www.editorialmanager.com/pbiology/> and log in as an Author. Click the link labelled 'Submissions Needing Revision' where you will find your submission record.

Thank you again for your submission to our journal. We hope that our editorial process has been constructive thus far, and we welcome your feedback at any time. Please don't hesitate to contact us if you have any questions or comments.

Sincerely,

Roli Roberts


Roland G Roberts, PhD,
Senior Editor
PLOS Biology

*****************************************************

REVIEWERS' COMMENTS:


Reviewer #1:

I enjoyed reading this paper, which was very well written and structured. The Introduction provided a concise but thorough overview of the current state of biologging technology, and clearly identified where the gap/unresolved issues are. The methods that are presented are novel and have the potential to offer a step-change in how movements of small animals can be studied.

We thank the reviewer for these supportive comments!

The authors do an excellent job of explaining why this novel system is so beneficial. I do think it would be useful also to have a greater discussion about what some of the potential drawbacks would be. In the Introduction the authors talk about how ICARUS and other existing techniques are limited, but then this is one pilot study, essentially, on one animal group (bats) – will this really work for other systems, and if not, why not, and what systems is it unlikely to be useful for. To this end, a summary table that perhaps proposes some different scenarios and animal groups coupled with how this technique could be applied, and whether it would work, would be beneficial to readers. So I do think the paper needs some discussion of potential negatives. For example, setting up such a system in the field will not be straightforward, and you will need some prior knowledge of the animals (of interest) movements to set things up. What are the costs involved? How user friendly is it? Many scientists who use biologging technology are conservationists, ecologists etc, and their background will be varied – how simple is this system to implement in reality?

We agree with the reviewer that a tabulated comparison will be very helpful to the readers to get an impression which technology is ideally suited for which use case. We created a detailed table which compares different systems for monitoring small vertebrates and which provide solutions for localization and encounter detection using different approaches. We also give detailed information on the functionalities, on strengths and limitations of all systems, and on the costs of our and other systems. Please see the image below for a downscaled version of the new table:

We further added additional text to the manuscript to address several points raised by the reviewer:

(i) We added text to the conclusions on the limitations of different wireless biologging networks. We also added information on strengths and limitations to the conclusions (starting line 469ff):

*“Similarly, we believe that wireless biologging networks like the one presented here will greatly benefit biologging of small animal species that move over smaller and more predictable spatial scales, especially inside habitats where signal transmission is constrained. However, prior knowledge on the study subjects is often useful to ensure individuals regularly visit tracking grids or come close to download stations. High-tech tracking solutions may also increase the workload for maintenance and post-processing and the equipment may be more vulnerable to external factors, such as increased humidity in tropical ecosystem. Initial purchase costs may also be higher compared to older systems, but this difference might be compensated by the gain in data quantity and quality, the ability to re-use components (e.g. mobile nodes), and the high degree of automation, which can in turn reduce labor costs.“*

(ii) We added text to the methods section on our motivations to test this system on bats. We believe that a system suitable to track bats is likely to also allow tracking of other taxa (lines 786ff):

*“In general, we chose to use bats to demonstrate the high performance of this system since small body mass, nocturnal activity (no sunlight exposure), and flight confront tracking systems with major challenges. A system that is capable of automated tracking of bats may be likely to work for tracking other small terrestrial taxa as well.“*

(iii) We added information that this technology is indeed more laborious than other systems and give information how fast a tracking grid might be set up (see also limitations in the new Table 2; lines 475ff):

“*High-tech tracking solutions may also increase the workload for maintenance and post-processing and the equipment may be more vulnerable to external factors, such as increased humidity in tropical ecosystem.”*

(iv) We give details on the work required to set up the tracking grid (lines 244f):

*“For example, a setup as described above consisting of 11 localization nodes is deployed and configured by two people in one to two workdays.“*

I think the authors need to enlarge slightly their discussion about the work that originally took place in Panama, using – to some extent – a similar approach. What really didn’t work there, and what has been the massive step change with this method that permits such a system to now work.

We agree that those are relevant facts for the reader. We added information to the discussion on how this dramatic step forward was possible. The key advance was ultra-low power operation and communication, enabled by smart hardware (e.g., wake-up receiver) and sophisticated communication protocols (e.g. erasure coding). We highlight this by adding the direct comparison to EncounterNet as suggested by reviewer 3 (for text added to the discussion see section responding to reviewer 3).

We also added information on our Panama fieldwork, the limitations of our setup, and the problems that may arise in using high-tech in tropical environments (see cited text under (i) above).

As an end user of biologging technology, rather than someone involved in its design, the methods did make sense to me and the approach logical and correct, but a technical engineer might be better placed to double check the coding details behind the logging technology.

Minor – weight should be mass throughout;

We corrected weight to mass throughout.

line 59, energy may be better phrased as power?

Done.

Here also, vertebrates – could be invertebrates too, given the size of some of them

In theory yes; however, we refer to a comprehensive review here, which focuses on vertebrate biologging, so we chose not to mention invertebrates here.


Reviewer #2:

The manuscript provides a really interesting system for advanced, fine resolution tracking of multiple organisms. The contact data generated from the study system was very impressive. The concept of contact information collected and distributed described here is similar to several other approaches that have been described (e.g. zebraNet). It was not clear how novel and different the current system is relative to these other approaches. I would also like to see a better discussion of the costs of building and maintaining the infrastructure for the BATS system – a number of approaches have been proposed and used using local area data transmission through radio frequencies received through towers. How does this differ? How does it compare to other solutions in terms of cost? Without this information it is difficult to gauge the novelty and potential for the BATS system. The reason the ICARUS and GPS systems are alluring is the lack of infrastructure needed to deploy collars in
such systems.

We entirely agree (also see comments above). We now included a tabulated comparison to show the strengths and limitations, the functionalities and costs of the proposed system and other systems. There are indeed many systems out there so we therefore limit our comparison to systems that are suitable for tracking small animals (tags that weigh around 2.5 g and less). Note that ICARUS (mobile node 5g) and GPS systems with remote download are not available in this size class. We included smaller GPS loggers, which do have to be retrieved for data access (while those do not require additional ground infrastructure, all data are lost if the tag cannot be recovered – which happens frequently).

1.      The number of acronyms used in the main text and figure captions should be reduced to avoid reader confusion. Several were mentioned in the introduction, and then irregularly re-defined throughout the paper. For example MN is redefined in Figure 5 caption (Line 296), but not in Table 1 (Line 278). I recommend removing WBN, RSS, MN, GN and RSSI, because of their sporadic use throughout the paper.

We agree that the use of multiple acronyms is not appealing. We removed RSS, MN, GN and RSSI across the entire main text. We only decided to keep RSSI in Figure 1 (but removed all ‘GN’ and ‘MN’). However, we decided to keep WBN since ‘wireless biologging networks’ is a new term and we believe it is important to introduce it along with its abbreviation (which is equivalent to WSN = wireless sensor networks).

2.      You acknowledge that there is no single best method for animal tracking, and review the limitations and strengths of several tracking technologies in the context of different ecological phenomenon. It would also be useful to see a cost comparison of the high-resolution tracking solutions. At the very least, and mention of the costs of each of the tracking systems used in this paper would be helpful to understand where the BATS system sits relative to other lower-resolution tracking options.

This is a good point! We included information on the costs in detail in our tabulated comparison.

120: It was unclear what the long-range telemetry in Figure 1 was used for in the tracking system. On line 107 the synchronization of clocks is mentioned. Is that the core use of the long-range telemetry, or is their other contact data being sent when animals are outside of the localization grid?

This is correct; we could also transmit other data types. We now clarify this in the methods section (lines 632ff):

*”These timestamps are crucial for post-processing of meetings because they allow accounting for clock drift on the mobile node (depending on the specific aim of the study other data types such as sensor data could be transmitted instead).”*

200: Can a start point be added for the tracks in (b) and (c)?

Yes, we added arrows indicating flight direction.

203: Should read (b) and (c).

Correct! It now read *‘(b, c)’.*

398: Change “will allow to study the” to “will allow study on the”

Done.


Reviewer #3:
[identifies himself as Josh Firth]

This manuscript provides a detailed and useful description of a wireless biologging network system and demonstrates how deploying this technology produces some amazing data detailing bat behaviour. It is very timely given the recent advances of (and demand for) animal tracking systems to provide such fine-scale data. For instance, I’m certain that the data here could be useful for addressing various questions regarding ecology within these bat social systems. The manuscript is very well written and formatted. My comments below are given in the following sections (1) Comments on potential considerations and changes regarding (1a) insights into biology (1b) comparison to other technologies and (1c) cost, and (2) Minor suggestions that may (or may not) be helpful to the authors.

(1) Considerations and potential changes

(1a) Insight into biology

(1ai) Although the manuscript does an excellent job of describing the technology and its direct uses, it would be very helpful if more explanation of the potential insights into biology that these new improvements could provide (especially insights that might not have been possible to obtain prior to this). For instance, much effort is much into describing, and quantifying improvements such as energy consumption, lifetime, resolution, distance verification, etc etc, but it would be great to know how each of these different improvements will be directly useful for answering different ecological questions. Currently, readers (especially those with little knowledge of uses of tracking technologies) may be left unsure about how scientifically useful these advances are, and which of the advance will be useful for what questions. It would be a shame to miss out on the opportunity here to really show how general and useful advancements in these technologies can be

The reviewer raises an important point here. Of course, we designed this biologging system with lots of biological questions in mind, some of which we already managed to answer, others still being worked on. To give the reader a detailed impression on what is now possible using this technology, we now include the following information, which is partly based on work that has been recently published. Regarding proximity sensing, we added (lines 179ff):

*“As an example, we have been able to gain a deeper understanding of the intrinsic and extrinsic factors influencing the stability of social relationships in vampire bats by combining captive experiments and proximity sensing in the wild [Ripperger et al. 2019 Current Biology]. Tracking associations at high temporal resolution in the wild allowed us to gather evidence for rare and cryptic events such as maternal guidance in noctule bats [Ripperger et al. 2019 Biology Letters] and unfamiliar kin recognition in vampire bats [Ripperger et al. 2019 BioRxiv].”*

For tracking, we highlight the option to include experimental triggers (lines 487f):

*“Experimental setups including automated triggers (e.g., acoustic playbacks or other sensory cues) can be integrated with direct proximity sensing, creating exciting research opportunities. “*

(1aii) Secondly, also in relation to this general point of providing insights into biology, it currently isn’t entirely clear why the different bat datasets are all used in different ways to look at different components of behaviour. Obviously one of the major ‘selling’ points of this technology is that is should provide great generality in what behaviours can be examined, but the current framework within the MS makes it appear that different datasets have to be used to look at different behaviours. Ideally, the MS would make the most of this amazing data across systems and show that each of the components of behaviour (social interactions, movement angles, distances) can be looked at within each of the systems (rather than separately as currently done).

However, perhaps it is the case that each of these aspects can be looked at in each system, but that this would be far too much extra work here. This is fair enough I think, but it would be good for the MS to clearly state this, and state why each aspect of behaviour was looked at within each dataset specifically here. This would also be a good opportunity to address ‘Comment 1ai’ , as then it could be made clear why biologists might want to know more about each of these behaviours (interactions, angels, distances), and why these are important to larger biological questions and why these questions were not easy to answer before. Finally, the bat data here looks excellent, I’m sure a few new insights are provided just by quantifying these systems in this way, it’d be very exciting to here them (or a hint of them) here.
see response to comment 1ai for our changes to explain the big questions which may be answered.

We are glad that the reviewer pointed out that it was not clear why we present datasets from multiple deployments. The reviewer is right that it actually would be possible to present the full functionality and all related data types from the same field study (for example from the Forchheim test). However, the reason why we chose different scenarios to present the different functionalities of the system is based on the species-specific behaviour of the bats. Below, we explain based on the three major functionalities of the system.

High-resolution tracking: We know that mouse-eared bats reliably return to the same couple of focal foraging areas night after night. Therefore, this species is ideally suited to obtain flight trajectories from the same individuals during several nights. In contrast, vampire bats may simply go wherever the cattle decide to sleep each night and noctule bats forage rather opportunistically – so in both cases we can’t equip a stable and predictable food patch with a tracking grid. This is why we invested the highest effort in estimating the tracking accuracy (Figure 3a) at our mouse-eared bat field site and we invested the highest effort in getting tracking data on mouse-eared bats (Figures 3b,c). We also find it most intuitive to present the calibration data and the actual bat trajectories from the same setup.

Proximity sensing: We decided to present the vampire social network dataset because it is the densest one and includes the highest number of tagged individuals both in total and inside the same roost. The mouse-eared bats were caught in the foraging area and roosted at different colonies during day so they reported only a few meetings; similarly, the 34 noctules yielded sparse networks because the bats were spread across a greater number of roosts). The high number of tagged vampire bats and their movements within a single big tree roost were ideal to highlight the social dynamics which become apparent only with a high-resolution dataset (Figure 2). We also added this detail to the main text starting line 169:

*“The typical approach for collecting social network data from bats has been to observe some or all of the bats within each roost in a sample of identified roosts each day, however these co-roosting associations (e.g. Wilkinson 1984 Nature, Wilkinson et al. 2019 BES) cannot detect social structure for individuals sharing the same single roost throughout a study.”*

Long-range telemetry: While we also ran this part of the system in Forchheim (but not in Panama) we chose to present the Berlin/noctule data because the Berlin setup was intended to actually quantify the precise transmission distances. Also, noctule bats frequently switch roosting sites and individuals may disappear for several days. The long-range system allowed us to still receive a minimum of crucial data while the bats remained at unknown locations.

We summarized our motivations in a paragraph within the methods section (lines 777ff):

*“The proximity sensing performance was best demonstrated in the vampire bats study since the highest number of individuals was tagged here and group cohesion was high – ideal circumstances to test system performance in the presence of a large number of tagged individuals. We demonstrated high-resolution tracking with mouse-eared bats since individuals of this species regularly revisit their foraging grounds and can therefore reliably be tracked within an area of 1-2 ha across multiple days. We assessed transmission distances of long-range telemetry in noctule bats, because they frequently switched roosts and long-range telemetry provided a valuable opportunity to obtain data from individuals that moved to unknown locations.“*

(1b) Other technologies

(1bi) A bit more direct qualitative comparison would be interesting to hear too. Currently, it is fairly heavily focused on comparing to GPS tracking. This comparison is a little bit one-dimensional as GPS are mainly only used by those desiring long distance tracking (and those less interested in other questions) – the specific device considered in the MS wouldn’t be used for for short distances. Nothing too much more is required, but it’d be interesting to here which currently-used technology is most like this one, how it compares to recent developments in lab-based system technologies (e.g. automated high-res tracking of barcodes in zebrafinch systems), how useful this technology might be for lab-based systems.

As suggested by all three reviewers we now provide a table which compares several relevant systems (including the zebra-finch barcodes) and highlights the strengths and limitations of all systems including the one presented here.

(1bii) Similarly, some more direct quantitative comparisons to other technologies would be really interesting to see (see point above). Ideally, if quantitative comparisons using simulations could be straight-forward and easy-to-implement then that would be very helpful indeed. For example, it would be nice to know how the results from this high-res tracking technology compares to another similar (but I’m assuming lower-res) technology such as encounternet. Given the sampling rate here, it would be great (and fairly easy?) to resample the data from this technology at a rate equivalent to something like encounternet, and then see how much this changes the inferences about social interactions for instance?

We agree that quantitative comparisons will be very interesting to the reader. We therefore included diverse information on sampling rates and data quality in our newly created Table 2 so that a reader can instantly assess the quality of the data, which is provided by the respective systems.

While resampling our data based on the EncounterNet parameter settings appears very intuitive at first, it is actually a bit misleading since these are soft settings, which will always be tailored to a particular use case. We therefore chose a similar but slightly different qualitative comparison at first and then highlight what we would have missed in an earlier study using the low EncounterNet sampling rate. (1) We calculate the runtime of our 1g node based on the sampling intervals of the EncounterNet 1.3 g node, which can be easily taken from Table 1: EncounterNet in Levin et al. 2016 sampled at 20 s intervals. Our active/interactive interval-setting of 10/30 s is equivalent since active/passive rates were almost 50/50. While the intervals are very similar, our system achieves a runtime that is 20 times higher than EncounterNet (15h vs. 321h, see below). (2) We refer to a study where we detected a novel social behaviour in bats from evidence based on many short encounters, which might have stayed undetected at sampling rates of 20s (lines 417ff).

*‘We show that the use of wake-up receivers and adaptive operation paired with novel wireless communication protocols dramatically reduce the energy demand of such wireless sensor tags. Levin et al. achieve ca. 15 h logging time at 20 s inter-pulse intervals using a 1.3 g mobile node, which is similar to our simulated scenario of 10/30 s active/inactive inter-beacon intervals, which allows for a sampling period of 321 h using a 1 g mobile node. Extending inter-beacon intervals for the sake of prolonged runtimes always bears the risk of missing short but biologically relevant encounters. For example, at a 20 s inter-beacon interval we would have missed many encounters among flying bats, which were crucial to reveal evidence for maternal guidance in noctule bats [Ripperger et al. 2019 Biology Letters].’*

(1c) Cost

(1ci) The price (cost) of new technologies is a major barrier to many tracking applications, and this study acknowledges the general importance of cost in the discussion L342-347. But, I think it also needs to specifically state the cost of these devices clearly. I think this will be the main question on interested readers’ lips as they hear about this system (it was certainly a burning question for me when I was reading it!). Obviously animal behaviour research funds are often difficult to obtain in most countries, so cost will be the main factor for driving international interest in this.

We agree, this information is now given in the tabulated comparison.

(1cii) Along with raw cost of this set-up, some direct demonstration/discussion of cost-effectiveness would be great too. Obviously the system is technically superior to many current options in terms of performance, but how does it compare when cost effectiveness is considered? For instance, there are genuinely very-low cost animal tracking options out there now, with possibilities of building RFID recording devices and tags for 100individuals or so for total cost of around £100. I obviously don’t expect this new system to beat this kind of raw cost, but could it be potentially considered as more ‘cost effective’ than this given the exceptional data in produces?

We included the costs across systems in the tabulated comparison to show where the proposed system sits compared to other systems. We also added text to the conclusions that initial costs for such sophisticated systems might be much higher than for older technologies, however, tags might be refurbished which lowers subsequent costs. We do believe that the dramatic increase in data quality is worth the extra expense (lines 478ff).

*“Initial purchase costs may also be higher compared to older systems, but this difference might be compensated by the gain in data quantity and quality, the ability to re-use components (e.g. mobile nodes), and the high degree of automation, which can in turn reduce labour costs.“*

(2) Minor suggestions
The following comments might not need to be addressed (or even responded to individually) but I just thought I’d point out a few thoughts I had while reading the MS in case it is helpful.

(2a) I really like figure 1 – I think it looks great. But, what is the meaning of the placement of the trees? I initially thought they were referring to physical barriers and I was trying to determine what the presence/absence of trees in different places meant. But, I now think it’s just to show environment in general and the actual placement has no meaning? If the latter is correct, I suggest having them as background rather than objects.

Yes, the trees simply suggested that the setup was located in a natural environment. We chose to remove the trees and to slightly reorganize the items in the figure to meet the formatting requirements and also to remove almost all acronyms in Figure 1.

(2b) I work on using technologies for experiments, not just observations, so I was wondering whether this WBN set-up could be used to manipulate individuals automatically at all? If it can’t be used to carry out automated treatments itself, could it potentially be combined with other low-cost technologies that can e.g. RFID devices used to change predation preception (e.g. Voelkl et al 2016 Sci Reports) or control access to resources (Firth & Sheldon 2015 PRSB)? It’d be exciting to hear some exploratory ‘first-thoughts’ on this topic.

This is indeed possible and we indeed planned to implement experimental triggers. However, we did not do so, yet. We mention this exciting possible future opportunity in the conclusions (line 487):

*‘Experimental setups including automated triggers (e.g., acoustic playbacks or other sensory cues) can be integrated and direct proximity sensing will create exciting research opportunities.’*

(2c) I get why the acronym BATS was chosen, but I’m not sure it is the best for describing this system. Indeed, lots of different tracking systems are ‘broadly applicable’, so it might be better to have something that describes this tracking system? Also, as this study uses bats (actual bats) as the study system for this tracking technology, this acronym might not end up being the best for quickly representing the ‘broadly applicable’ aspect of this tech (runs the risk of people thinking its focused on bats!).

We agree. We decided not to use the long version of the acronym BATS ‘broadly applicable tracking system’ in the revised manuscript and we also avoided to use ‘BATS’ throughout the manuscript (see detailed answer in our comment above at the beginning of the manuscript).

L310 states ‘experimental design?’ Should this be ‘observational design’?

‘Experimental’ is indeed misleading. We now only say:

*‘the model is a reliable tool for designing a field study’*

L313-315 the discussion goes straight into statements about the technology, but ideally it would start with something biologically relevant (i.e.point 1a)

We appreciate this comment. In revising the manuscript, we thought through the central theme of our discussion, and how best to structure it. We believe it is a good fit for this methods paper to start with a discussion of the technology first and then end more broadly with the outlook and the biological applications. We appreciate the reviewer’s suggestion but feel that the current organization works well to avoid redundancy. We are happy to change this structure however if so advised; we leave this decision to the editor’s discretion.

I hope these comments are useful to the authors for this excellently coherent and interesting description of this exciting new technology
Josh Firth (please note, I sign all my reviews).
