# Supplementary material for: Thinking small: Next-generation sensor networks close the size gap in vertebrate biologging
Source: PLoS Biol. 2020 Apr 2;18(4):e3000655. doi: 10.1371/journal.pbio.3000655 (PMC7117662; doi:10.1371/journal.pbio.3000655)
Supplement: Attachment — Submitted filename: response to editorial comments.docx [file pbio.3000655.s006.docx]

Dear Dr Ripperger,

Thank you for submitting your revised Methods and Resources paper entitled "Thinking small: next-generation Sensor Networks close the Size Gap in Vertebrate Biologging" for publication in PLOS Biology. The Academic Editor and I have now assessed your revisions.

Based on this assessment, we will probably accept this manuscript for publication, assuming that you will modify the manuscript to address the following points:

IMPORTANT: We and the Academic Editor are concerned that your manuscript currently gives insufficient detail for readers to replicate your Method. This is particularly crucial for a Methods and Resources paper, and in order to comply with PLOS' policy on reproducibility. We therefore request that you supply, preferably as Supplementary files, clearly cited in your Methods section (and/or elsewhere in the manuscript) the following:

We chose a combination of supplementary information and GFBio depositions for providing all required information (see details below). We link to our data repositories or the supplement in the legends of our figures and in the methods section, e.g.:

Line 535 tracked changes manuscript: *(software and hardware design can be downloaded at* [*http://coll.mfn-berlin.de/data/10.7479/z5ym-kx58*](http://coll.mfn-berlin.de/data/10.7479/z5ym-kx58)*).*

Legend Fig 2*: Data and software used to create this figure have been archived by GFBio (*[*https://doi.org/10.7479/vd6t-7a92*](https://doi.org/10.7479/vd6t-7a92)*, https://doi.org/10.7479/ytdf-wf05).*

a) Circuit diagram (and preferably the printed circuit design), parts list (with costings, if possible), assembly instructions, 3D printing file (STL) for the case, and description of means of attachment, for the mobile node.

We added a diagram and the STL for the housing as supplements, including a parts list for the major components and assembly instructions. Please note that we decided not to provide pricing for the individual items. Basically all components are produced in China and therefore underlie strong fluctuations in availability and price based on the geopolitical situation. Googling the individual items on the parts list will give up-to-the-minute pricing.

Please also note that we decided to add a block diagram (plus parts list) rather than a circuit diagram since circuit diagrams quickly become outdated. The circuit design for the mobile node used in our study is already becoming outdated for the following reasons:

* two major components in our design are currently already becoming unavailable (e.g., the 2.4GHz chip antenna by AMCA31-2R450G-S1F-T von Abracon) or production has stopped and clearance sale has started (HF-switch SKY13350 by Skywork Solutions Inc.). This means that within a few months, the current design cannot be built anymore and a minor re-design will be required (swapping aforementioned parts plus periphery for follow-up models).

* passive components (e.g., resistors, inductors or capacitors which are not shown in the block diagram, but would be shown in a circuit diagram) are particularly affected by the current market situation, not only in terms of pricing but also in terms of availability. Therefore, these parts always need to be selected based on the current availability on the market.

* During the course of our project, we had to swap several smaller components for subsequent sensor node batches based on current availability. For example, a quartz crystal (CX2016DB by Kyocera) and the DC-to-DC converter (TPS82740) became temporarily unavailable (for 6 months in case of the quartz). Once these parts must be replaced, the periphery must be modified as well.

* ‘Smaller’ components such as capacitors depend upon the ‘larger’ components, which come with data sheets containing recommendations for the periphery. These recommendations keep changing, e.g. for the EFR32, Silicon Labs now recommends a 4.7µF capacitor, while we used a formerly recommended 1µF capacitor. Therefore, our current design is not in line with the manufacturer’s recommendations anymore. It is therefore always recommended to re-visit the data sheets and update the design according to the latest recommendations prior to each production round.

b) Code for the Raspberry Pi at the ground node, plus other details for constructing the ground node, including SD card, Wifi, housing (STLs?), etc.

We created another data deposition on GFBio (<http://coll.mfn-berlin.de/data/10.7479/z5ym-kx58>) containing the software for the ground node (two separate images for the Raspberry Pi and the STM board) and a description of all hardware parts and illustrated assembly instructions (Readme file). Note that no STL is required in this case. The ground node is housed in a standard plastic box from a hardware store.

c) In previous analogous instances, we have recommended that authors protect their hardware design using an Open Source Hardware, along the same lines as the CC-BY used for PLOS content: <http://freedomdefined.org/OSHW> - there are similar licences for software. We suggest that you do this.

We protect our GFBio depositions accordingly (e.g., GPL v3).

d) Additional numerical data underlying the Figures (see Data Policy request below)
see response below.

We expect to receive your revised manuscript within two weeks. Your revisions should address the specific points made by each reviewer. In addition to the remaining revisions and before we will be able to formally accept your manuscript and consider it "in press", we also need to ensure that your article conforms to our guidelines. A member of our team will be in touch shortly with a set of requests. As we can't proceed until these requirements are met, your swift response will help prevent delays to publication.

We changed the manuscript according to the formatting requirements provided by your team.

------------------------------------------------------------------------
ETHICS STATEMENT:

The Ethics Statements in the submission form and Methods section of your manuscript should match verbatim. Please ensure that any changes are made to both versions.

We matched the verbatim of the manuscript and the submission form.

-- Please include the full name of the IACUC/ethics committee that reviewed and approved the animal care and use protocol/permit/project license. Please also include an approval number.

We now include the full names of the approving institutions and all approval numbers.

*Work on vampire bats: All experiments were approved by the Smithsonian Tropical Research Institute Animal Care and Use Committee (#2015-0915-2018-A9 and #2017-0102-2020) and by the Panamanian Ministry of the Environment (#SE/A-76-16 and #SE/AH-2-17).*

*Work on noctule bats: All necessary permits were obtained from ‘Senatsverwaltung für Umwelt, Verkehr und Klimaschutz’ in Berlin (I E 222/OA-AS/G_1203) and ‘*[*Landesamt für Gesundheit und Soziales*](https://www.berlin.de/lageso/)*’ (I C 113-G0008/16).*

*Work on mouse-eared bats: All experiments were approved by the government of Upper Franconia (55.1-8642.01-15/13) and by the government of Lower Franconia (55.2-DMS-2532-2-181).*

-- Please include the specific national or international regulations/guidelines to which your animal care and use protocol adhered. Please note that institutional or accreditation organization guidelines (such as AAALAC) do not meet this requirement.

We included these details:

(1) Panama:

*Our protocols adhered to the following guidelines: (1) The U.S. Government Principles for the Utilization and Care of Vertebrate Animals Used in Testing, Research, and Training, developed by the Interagency Research Animal Committee and adopted in 1985 by the Office of Science and Technology Policy; (2) The Animal Welfare Act, 7 United States Code (U.S.C.) §2131 et. seq., and the regulations promulgated thereunder by the U.S. Department of Agriculture (USDA); and (3) Public Health Service (PHS) Policy on Humane Care and the Use of Laboratory Animals, August 2002, for all PHS- or National Science Foundation (NSF)-supported activities involving vertebrate animals.*

(2) Germany:

*Our protocols adhered to the following guidelines: (1) The Directive 2010/63/EU of the European Parliament and of the Council of 22 September 2010 on the protection of animals used for scientific purposes; (2) Bundesnaturschutzgesetz (BNatSchG); (3) Tierschutzgesetz (TierSchG); and (4) Verordnung zum Schutz von zu Versuchszwecken oder zu anderen wissenschaftlichen Zwecken verwendeten Tieren (Tierschutz-Versuchstierverordnung - TierSchVersV).*

-- Please include information about the form of consent (written/oral) given for research involving human participants. All research involving human participants must have been approved by the authors' Institutional Review Board (IRB) or an equivalent committee, and all clinical investigation must have been conducted according to the principles expressed in the Declaration

No research involving human participants has been conducted.

------------------------------------------------------------------------

DATA POLICY:

You may be aware of the PLOS Data Policy, which requires that all data be made available without restriction: <http://journals.plos.org/plosbiology/s/data-availability>. For more information, please also see this editorial: <http://dx.doi.org/10.1371/journal.pbio.1001797>

We note that the raw tracking data and analysis code are provided in the GFBio depositions. However, we also require the numerical values that directly underlie your Figure to be made available in one of the following forms:

The data underlying the proximity/tracking figures (Fig 2 and 3) are already included in our GFBio deposition, which is now accessible online (<http://coll.mfn-berlin.de/data/10.7479/vd6t-7a92>; datasets 1 and 2 – note that dataset 2 includes a description of the file inventory as a separate download). The deposition contains the code and data to construct the trajectories in figure 3. We added dataset 4 and 5 to the repository, which underlie figure 4 and figure 5 of the manuscript (see details below).

1) Supplementary files (e.g., excel). Please ensure that all data files are uploaded as 'Supporting Information' and are invariably referred to (in the manuscript, figure legends, and the Description field when uploading your files) using the following format verbatim: S1 Data, S2 Data, etc. Multiple panels of a single or even several figures can be included as multiple sheets in one excel file that is saved using exactly the following convention: S1_Data.xlsx (using an underscore).

We added the mobile node design to the supplement (S2 Fig + S1 Data + detailed legend including parts list and assembling instructions).

2) Deposition in a publicly available repository. Please also provide the accession code or a reviewer link so that we may view your data before publication.

Our data repositories at GFBio are now public and can be accessed without restriction.

Regardless of the method selected, please ensure that you provide the individual numerical values that underlie the summary data displayed in the following figure panels as they are essential for readers to assess your analysis and to reproduce it: Figs 4 and 5 (I am assuming that Figs 2 and 3 can be plotted fairly directly from the GFBio tracking data). NOTE: the numerical data provided should include all replicates AND the way in which the plotted mean and errors were derived (it should not present only the mean/average values).

Yes, Fig 2 and 3 can be plotted directly from the GFBio deposition. Figure 4 as well, code and raw data have now been added to the existing deposition (<http://coll.mfn-berlin.de/data/10.7479/vd6t-7a92>; datasets 2 (code) & 4 (raw data)). In addition, we added the raw data underlying Figure 5 to the existing GFBio deposition (Dataset 5). We created a new GFBio deposition including hardware/software for the ground node (<http://coll.mfn-berlin.de/data/10.7479/z5ym-kx58>).

Please also ensure that figure legends in your manuscript include information on where the underlying data can be found (including GFBio URLs/DOIs), and ensure your supplemental data file/s has a legend.

We added DOIs to all figure legends.

Please ensure that your Data Statement in the submission system accurately describes where your data can be found.

We provide the detailed citations of our data publications as recommended by GFBio (including DOIs).
